# Dr. John Preston

**Published:** 1891-02

**Authors:** 


Dr. John Preston, the new superintendent of the State Lunatic Asylum at Terrell, was born in Washington county, Va., July 12, 1851; was educated by private teachers; afterwards at Georgetown college; received medical education at the University of Virginia in 1872-3, and at Bellevue, New York, in 1873-4, graduating at each. He was offered the appointment of assistant physician in the New York Insane Asylum on Blackwell's Island just after graduating at Bellevue, but declined, and entered on the practice of his profession at Aldie, Va. Remained there six months, and removed to Texas, and located at Seguin. Returned to Virginia and located near Bristol. In 1878 returned to Seguin, and built up a good practice. February, 1886, was appointed First Assistant Physician of the State Lunatic Asylum. Resigned in September, 1890, and entered upon general practice at San Antonio. Elected Superintendent of the North Texas Insane Asylum February 10, 1891.

